# Ancient Monuments of the Mississippi Valley

**Published:** 1849-01

**Authors:** 


### Article VII.—*Ancient Monuments of the Mississippi Valley.*

This great American work, the result of original surveys and explorations by Messrs. E. G. Squier and E. H. Davis, has the honor of being selected as the first volume of the "Smithsonian Contributions to Knowledge," published by the Smithsonian Institution. It will contain about six hundred pages of letter-press, in size and style uniform with the quarto edition of the "United States' Exploring Expedition," and illustrated by upwards of fifty quarto plates, and more than two hundred engravings on wood. It embraces plans from actual surveys of more than one hundred and fifty ancient earth and stone works, with plans, sections, and views illustrative of the present appearance, position, structure, contents, and purposes of the several varieties of aboriginal mounds and pyramids; also sketches and notices of the minor vestiges of ancient art—the implements, weapons, ornaments, sculptures, &c., found in the mounds.—*Bos. Med. and Surg. Jour.*

